# *Notes from the Field:* Contact Tracing for *Monkeypox virus* Clade I Cases Associated with Air Travel — United States, November 2024–January 2025

**DOI:** 10.15585/mmwr.mm7515a2

**Published:** 2026-04-23

**Authors:** Roberta Lugo Robles, Alida M. Gertz, Francisco Alvarado-Ramy, Adelaide Asare, Kristen Pringle, Kara Adams, Yonette Hercules, Mayra Garcia Brown, Justice King, Linda C. Pimentel, Faisal S. Minhaj, Clive Brown, Sundari Mase, Shannon Gearhart

**Affiliations:** ^1^Division of Global Migration Health, National Center for Emerging Zoonotic and Infectious Diseases, CDC; ^2^Cherokee Nation Operational Solutions, Catoosa, Oklahoma; ^3^Division of High-Consequence Pathogens and Pathology, National Center for Emerging Zoonotic and Infectious Diseases, CDC.

**Summary**

**What is already known about this topic?**

Aircraft contact investigations of persons traveling with clade II *Monkeypox virus* (MPXV) infection suggest that clade II MPXV transmission risk during commercial air travel is low; however, data indicate that clade I MPXV might be more transmissible than clade II.

**What is added by this report?**

Among 60 aircraft contacts of three persons with laboratory-confirmed clade I MPXV who traveled on five commercial flights and were investigated to assess transmission risk during air travel, no secondary cases were identified. CDC discontinued aircraft contact investigations for all MPXV clades and subclades in 2025.

**What are the implications for public health practice?**

Similar to clade II, clade I MPXV transmission risk during commercial air travel appears to be low.

## Introduction

Monkeypox is a vesicular rash illness caused by *Monkeypox virus* (MPXV), which can be divided into clades (e.g., clade I and II), each of which includes subclades. MPXV is usually transmitted from person to person through close, sustained physical contact. Global spread of clade II MPXV began in 2022; to date, approximately 37,500 cases have been reported in the United States (provisional data), and domestic transmission is ongoing. CDC began conducting aircraft contact investigations for clade II MPXV in 2021. In February 2023, based on data showing no evidence of in-flight transmission of clade II MPXV, CDC discontinued routine aircraft contact investigations. Although outcome data were missing for approximately one third of identified aircraft contacts, a 2024 report describing data from 2021 to 2022 on clade II MPXV transmission risk during commercial air travel found no secondary cases reported among 1,538 persons who had contact with 113 infected travelers on 221 flights (*1*).

Outbreaks caused by clade I MPXV started in Central Africa in 2023 and 2024 and spread across the African continent, resulting in approximately 150 travel-associated cases outside of Africa. Limited data suggested that clade I MPXV might be more transmissible than clade II (*2*). Historically, clade I MPXV outbreaks have resulted in higher case-fatality rates (CFRs) than did those associated with clade II MPXV infections (*3*); however, recent clade I outbreaks have been associated with lower CFRs than those historically reported, with some studies describing CFRs similar to those associated with clade II infections (*4*). Investigations of clade I MPXV transmission risk during commercial air travel are limited. This report describes the results of an analysis of aircraft contact investigations after identification of three travelers who flew on commercial aircraft into or within the United States while infectious with clade I MPXV.

## Investigation and Outcomes

### Data Source

During November 2024–January 2025, CDC was notified by U.S. health departments and one foreign health authority of three men aged 20–40 years who traveled on five flights while infectious* with confirmed clade I MPXV. Each of the three passengers traveled separately on international flights inbound to the United States, and two took subsequent domestic flights. Data from aircraft contact investigations coordinated by CDC were analyzed. This activity was reviewed by CDC, deemed not research, and conducted consistent with applicable federal law and CDC policy.^†^

### Characteristics of Infected Travelers

Signs and symptoms of MPXV reported by the three infected passengers during travel included skin lesions or rash (two), fever (one), and malaise (one). Additional signs and symptoms documented after arrival included fatigue (one), nausea (one), inguinal lymphadenopathy (one), and an ulcerative lesion in the genital region (one).

### Identification and Characteristics of Aircraft Contacts

Air travel contacts were defined as crew members who served the infected travelers and passengers seated within two seats (6 ft [1.8 m]) of the infected travelers. Eighty contacts were identified, all of whom were designated as having uncertain to minimal risk.^§^ The number of identified contacts on each flight ranged from nine to 24. Twenty contacts were excluded from further analysis for the following reasons: 1) 11 (14%) had known contact (e.g., travel companion or household member) with the infected passenger outside of the flight, 2) five (6%) had already departed the United States at the time of the investigation, and 3) four (5%) did not have a U.S. address (i.e., incomplete location information or no U.S. address available to CDC). The 60 (75%) remaining contacts for whom CDC sent notifications to state or territorial health departments were included in this analysis (Table). Of those, women accounted for 57% of identified contacts, and the median age was 43 years (IQR = 30–57 years). Overall, health departments succeeded in interviewing 29 (48%) contacts. One contact reported fatigue, body aches, and a skin lesion after the flight; subsequent polymerase chain reaction testing of the lesion was negative for MPXV. No secondary cases were identified.

**TABLE:** Characteristics of contacts identified on five commercial flights,* each carrying a passenger^†^ with clade I *Monkeypox virus* infection — United States, November 2024–January 2025

Flight characteristic	Flight duration, hrs	No. (%)			
		CDC notification sent to state and territorial health departments, no. of contacts	Contacts interviewed^§^	Female sex	Median age, yrs (IQR)
Flight 1: international	≥8	10	4 (40)	6 (60)	39 (31–60)
Flight 2: international	≥8	10	4 (40)	6 (60)	38 (20–56)
Flight 3: domestic	<4	15	6 (40)	10 (67)	36 (26–52)
Flight 4: domestic	<4	9	6 (67)	4 (44)	37 (27–52)
Flight 5: international	≥8	16	9 (56)	8 (50)	51 (45–59)
**Total**	**NA**	**60**	**29 (48)**	**34 (57)**	**43 (30–57)**

**Abbreviations:** MPXV = *Monkeypox virus*; NA = not applicable.

* Listed in chronological order of occurrence.

**^†^** Three men aged 20–40 years traveled on five separate flights while infectious with confirmed clade I MPXV infection. Each of the three passengers traveled on an international flight inbound to the United States, and two took subsequent domestic flights.

^§^ A total of 12 of the 29 interviewed contacts reported no history of vaccination. Vaccination status for the other interviewed contacts was not reported.

## Preliminary Conclusions and Actions

Similar to results from clade II MPXV aircraft contact investigations (*1*), this analysis suggests that the risk for clade I MPXV transmission during commercial air travel is low; however, this analysis was limited by a small sample size and the low proportion (fewer than one half) of contacts interviewed. Based on the absence of evidence for on-board transmission provided by these data, CDC discontinued aircraft contact investigations for all MPXV clades and subclades in 2025. Although evidence suggests a low risk for clade I and II MPXV transmission during air travel, persons with monkeypox are advised to delay travel until their illness has resolved. Persons traveling to destinations with outbreaks should be provided with information about the risks associated with monkeypox. Vaccination of groups at risk for monkeypox has been shown to be an effective public health mitigation measure and can help to decrease spread and disease severity (*5*).

## Footnotes

* For these contact investigations, the infectious period was defined as beginning with the onset of symptoms through the time all lesions had crusted over, the crusts had separated, and a fresh layer of healthy skin had formed under the crust. Clinical Signs and Symptoms of Monkeypox | Monkeypox | CDC

† 45 C.F.R. part 46.102(l)(2), 21 C.F.R. part 56; 42 U.S.C. Sect. 241(d); 5 U.S.C. Sect. 552a; 44 U.S.C. Sect. 3501 et seq.

§ Identification of contacts and assessment of exposure risk was based on modified CDC guidance. Risk Assessment and Monitoring in Community Settings | Monkeypox | CDC
